# Exercise Training for Blood Pressure: A Systematic Review and Meta‐analysis

**DOI:** 10.1161/JAHA.112.004473

**Published:** 2013-02-22

**Authors:** Veronique A. Cornelissen, Neil A. Smart

**Affiliations:** 1 Faculty of Kinesiology and Rehabilitation Sciences, KU Leuven, Leuven, Belgium (V.A.C., N.A.S.); 2 School of Science and Technology, University of New England, Armidale, 2351, New South Wales, Australia (N.A.S.)

**Keywords:** adults, blood pressure, exercise, humans, training

## Abstract

**Background:**

We conducted meta‐analyses examining the effects of endurance, dynamic resistance, combined endurance and resistance training, and isometric resistance training on resting blood pressure (BP) in adults. The aims were to quantify and compare BP changes for each training modality and identify patient subgroups exhibiting the largest BP changes.

**Methods and Results:**

Randomized controlled trials lasting ≥4 weeks investigating the effects of exercise on BP in healthy adults (age ≥18 years) and published in a peer‐reviewed journal up to February 2012 were included. Random effects models were used for analyses, with data reported as weighted means and 95% confidence interval. We included 93 trials, involving 105 endurance, 29 dynamic resistance, 14 combined, and 5 isometric resistance groups, totaling 5223 participants (3401 exercise and 1822 control). Systolic BP (SBP) was reduced after endurance (−3.5 mm Hg [confidence limits −4.6 to −2.3]), dynamic resistance (−1.8 mm Hg [−3.7 to −0.011]), and isometric resistance (−10.9 mm Hg [−14.5 to −7.4]) but not after combined training. Reductions in diastolic BP (DBP) were observed after endurance (−2.5 mm Hg [−3.2 to −1.7]), dynamic resistance (−3.2 mm Hg [−4.5 to −2.0]), isometric resistance (−6.2 mm Hg [−10.3 to −2.0]), and combined (−2.2 mm Hg [−3.9 to −0.48]) training. BP reductions after endurance training were greater (*P*<0.0001) in 26 study groups of hypertensive subjects (−8.3 [−10.7 to −6.0]/−5.2 [−6.8 to −3.4] mm Hg) than in 50 groups of prehypertensive subjects (−2.1 [−3.3 to −0.83]/−1.7 [−2.7 to −0.68]) and 29 groups of subjects with normal BP levels (−0.75 [−2.2 to +0.69]/−1.1 [−2.2 to −0.068]). BP reductions after dynamic resistance training were largest for prehypertensive participants (−4.0 [−7.4 to −0.5]/−3.8 [−5.7 to −1.9] mm Hg) compared with patients with hypertension or normal BP.

**Conclusion:**

Endurance, dynamic resistance, and isometric resistance training lower SBP and DBP, whereas combined training lowers only DBP. Data from a small number of isometric resistance training studies suggest this form of training has the potential for the largest reductions in SBP.

## Introduction

Current National Health and Nutrition Examination Survey data suggest that the prevalence of hypertension (HTN) varies with ethnicity and gender but lies between 25% and 43% in the US population, with an upward trend during the past 3 National Health and Nutrition Examination Surveys.^1^ HTN, or the chronic elevation of resting arterial blood pressure (BP) >140 mm Hg systolic (SBP) and/or 90 mm Hg diastolic BP (DBP), remains one of the most significant modifiable risk factors for cardiovascular disease (eg, coronary artery disease, stroke, heart failure).^2^ Although antihypertensive medications are efficacious and most have minimal side effects, the economic health care costs are increasing.^3^ Both national and international treatment guidelines for the primary and secondary prevention of HTN recommend nonpharmacological lifestyle modifications as the first line of therapy, including increasing levels of physical activity.^4^ There is Class I, Level B evidence that 150 minutes of weekly physical activity offers an alternative that may be used to complement antihypertensive medication.^5^

The American College of Sports Medicine position stand on exercise and HTN^6^ recommends dynamic aerobic endurance training for at least 30 minutes daily, preferably supplemented with dynamic resistance exercise. The effects of exercise training may vary with different exercise modalities (eg, endurance training or resistance exercise) and dose parameters, specifically program length, session duration, frequency, and workload or intensity. As such, the optimal exercise training prescription remains unclear. Dynamic aerobic endurance exercise involves large muscle groups in dynamic repetitive activities that result in substantial increases in heart rate and energy expenditure. Resistance training is activity in which each effort is performed against a specific opposing force generated by resistance and is designed specifically to increase muscular strength, power, and/or endurance. According to the type of muscle contraction, resistance training can be divided into 2 major subgroups: “dynamic” versus “static or isometric” resistance training. Dynamic resistance training involves concentric and/or eccentric contractions of muscles while both the length and the tension of the muscles change. Isometric exertion involves sustained contraction against an immovable load or resistance with no or minimal change in length of the involved muscle group. Current thinking varies with respect to the preferred type of physical activity for BP; historically endurance training has been preferred. Isometric activity has previously been associated with exaggerated hypertensive responses, but recent work has suggested isometric handgrip activity may become a new tool in the nonpharmacological treatment of high BP.^7–8^ Previous meta‐analyses have examined the effects of endurance training,^9^ dynamic resistance training,^10–11^ and isometric resistance training^7–8^ in isolation on BP, although a meta‐analytic comparison of all different exercise modalities, strictly limited to randomized controlled trials and eliminating data from crossover studies, has not been conducted.

The aims of this work were to (1) conduct a systematic review and meta‐analysis of randomized controlled trials to compare the effects of endurance training, dynamic resistance training, isometric resistance training, or combined endurance and resistance training on the magnitude of change in SBP and DBP in subclinical populations; (2) examine whether magnitude of change in SBP and DBP was different with respect to sex, age, and BP classification; and (3) examine whether magnitudes of change in SBP and DBP were related to exercise program characteristics, that is, program duration, exercise session duration, exercise intensity, exercise mode, weekly exercise duration, or weekly session frequency.

## Methods

### Search Strategy

A database of randomized controlled trials on the effect of exercise training on BP was started in 1985^12^ and updated in 1994,^13^ 1999,^14^ 2003,^9–10^ and again for the current meta‐analysis. Potential new studies were identified by a systematic review librarian. A systematic search was conducted of Medline (Ovid), Embase.com, and SportDiscus for the period November 1, 2003 until February 28, 2012. The search strategy included a mix of medical subject headings and free text terms for the key concepts aerobic/dynamic/endurance/resistance exercise, training, HTN, and SBP/DBP, and these were combined with a sensitive search strategy to identify randomized controlled trials. Reference lists of articles found were scrutinized for new references. The full search strategy for one of the databases (PubMed) is available on request of the corresponding author. No language limits were imposed.

### Inclusion Criteria

The inclusion criteria for this meta‐analysis were as follows: (1) randomized controlled parallel‐design trials of exercise training for a minimum of 4 weeks; (2) participants were adults (age ≥18 years) without cardiovascular or other diseases; (3) the study reported before and after mean and SD (or standard error) of resting BP in exercise and control groups or mean change and SD (or standard error) in exercise and control groups; and (4) the study was published in a peer‐reviewed journal up to February 2012. Any studies not meeting these criteria were excluded. All identified articles were assessed independently by 2 reviewers (N.A.S. and V.A.C.), and disagreements were resolved by discussion.

### Data Extraction

Data relating to subject characteristics, exercise program characteristics, and the primary outcomes were systematically reviewed. Information was archived independently in a database by each author. Discrepancies were resolved by consensus. Study quality was evaluated according to the Physiotherapy Evidence Database (PEDro) scale.^15^ However, we regarded participant and therapist blinding and allocation concealment as practical, so the maximum number of points possible was 8. Further, BP measurements using an automated, semiautomated, or random‐zero device were considered as investigator blinded measurements.

### Statistical Analysis

All meta‐analyses were performed using Comprehensive Meta Analysis (CMA) V2 software (Biostat, NJ). The primary outcome measures were changes in resting SBP and DBP. Descriptive data of treatment groups and participants are reported as the mean±SD or median and range. Effect sizes for each study group were calculated by subtracting the preexercise value from the postexercise value (post–pre) for both the exercise (Δ1) and control groups (Δ2). The net treatment effect was then obtained as Δ1 minus Δ2. Variances were calculated from the pooled SDs of change scores in the intervention and control groups. If change score SDs were not available, these were calculated from pre‐SD and post‐SD values for which a correlation coefficient of 0.5 between the initial and final values was assumed.^16^ Each effect size was then weighted by the inverse of its variance. Random‐effects models that incorporate heterogeneity into the model were used to pool all primary and secondary outcomes from each study group.

Statistical heterogeneity among the studies was assessed using Cochran Q test, with a *P*>0.05 considered statistically significant and an inconsistency *I*² statistic in which a value >50% was considered indicative of high heterogeneity. Four main comparisons were made with each exercise group being compared with a no‐intervention (sedentary) control group: that is, endurance training, dynamic resistance training, combined training, and isometric resistance training. In addition, a fifth comparison between endurance training and dynamic resistance training was made including trials that involved both an endurance training and dynamic resistance training arm. If trials compared multiple exercise interventions with a single control group within one comparison, we split the shared control group into ≥2 groups with smaller sample size.^17^ We used a 5% level of significance and 95% CIs for all outcomes.

Using stratified meta‐analyses, we tested 8 a priori hypotheses that there may be differences in the effect on BP with regard to type of exercise (endurance training, dynamic resistance training, combined training, isometric resistance training) and for endurance training and dynamic resistance training across particular subgroups, sex (men versus women), age (<50 versus ≥50 years), weekly frequency, training intensity, session duration (minutes), program duration (weeks), BP classification using the Seventh Report of the Joint National Committee on Prevention, Detection, Evaluation, and Treatment of High Blood Pressure,^4^ and total weekly exercise time. *Z* tests were used to compare summary variables.

In addition, simple random‐effects meta‐regression analysis (methods of moment approach) was performed to investigate the association between changes in BP and changes in weight.

Finally, to qualitatively assess publication bias, funnel plots of the effect size versus the standard error AUTHOR: Does standard error refer to SEM or SEE?for each study group were generated. Funnel plot asymmetry was evaluated by use of Begg and Egger tests, and a significant publication bias was considered if the *P* value was <0.10.^18^ The trim and fill computation was used to estimate the effect of publication biases on the interpretation of the results.^18^ Cumulative meta‐analyses, ranked by year, were used to examine results over time for each of the different training modalities.

## Results

### Literature Search

**Figure 1.**
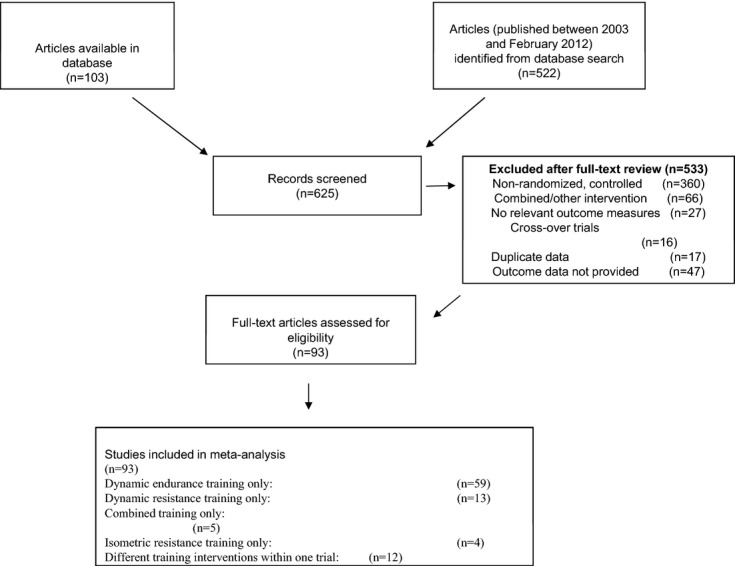
PRISMA flow diagram. PRISMA indicates preferred reporting items for systematic reviews and meta‐analyses.

One hundred three articles published between 1976 and 2003 were already available in our database as they were used for previous reviews. The electronic search yielded an additional 522 citations, which were screened by reviewing the title or abstract of each. Of these 625 publications, 93 trials were included in the meta‐analysis (Figure 1). Some of these trials involved several groups of individuals or applied different training regimens, so that a total of 153 study groups (ie, 105 endurance training, 29 dynamic resistance training, 5 isometric resistance training, and 14 combined training groups) were available for meta‐analysis. A general description of each trial is shown in Table S1. The studies enrolled 5223 patients: 3401 were exercise training participants and 1822 were sedentary controls. Based on the average baseline BP, 47 study groups included individuals with normal BP (29 endurance training, 12 dynamic resistance training, 2 isometric resistance training, and 4 combined), 73 study groups involved prehypertensive participants (50 endurance training, 13 dynamic resistance training, 2 isometric resistance training, and 8 combined training), and 33 training interventions were performed in hypertensive patients (26 endurance training, 4 dynamic resistance training, 1 isometric resistance training, and 2 combined training).

Collectively, exercise intervention length ranged from 4 to 52 weeks. For those studies that reported data, the between‐study frequency ranged from 1 to 7 times per week, and intensity ranged from 35% to 95% peak oxygen consumption for endurance training, between 30% and 100% of 1‐repetition maximum for dynamic resistance training, and between 10% and 40% for isometric resistance training.

Study quality is summarized in Table S2. The median PeDro score was 6 of 8. Ninety (97%) trials clearly stated eligibility criteria, all studies were randomized, and 90 (97%) studies matched intervention groups at baseline for BP, although groups were also well matched for age and sex. Blinding of outcome assessment was performed in 58 (62%) studies, but no more than 8 trials specifically reported that the observers were blinded to treatment allocation. Only 44 (47%) of studies clearly reported that >85% of participants had complied with the intervention, only 7 (8%) studies completed an intent‐to‐treat analysis, 90 (97%) studies completed between‐group analyses, and all studies provided point estimates for effect size.

### Quantitative Data Synthesis

**Figure 2.**
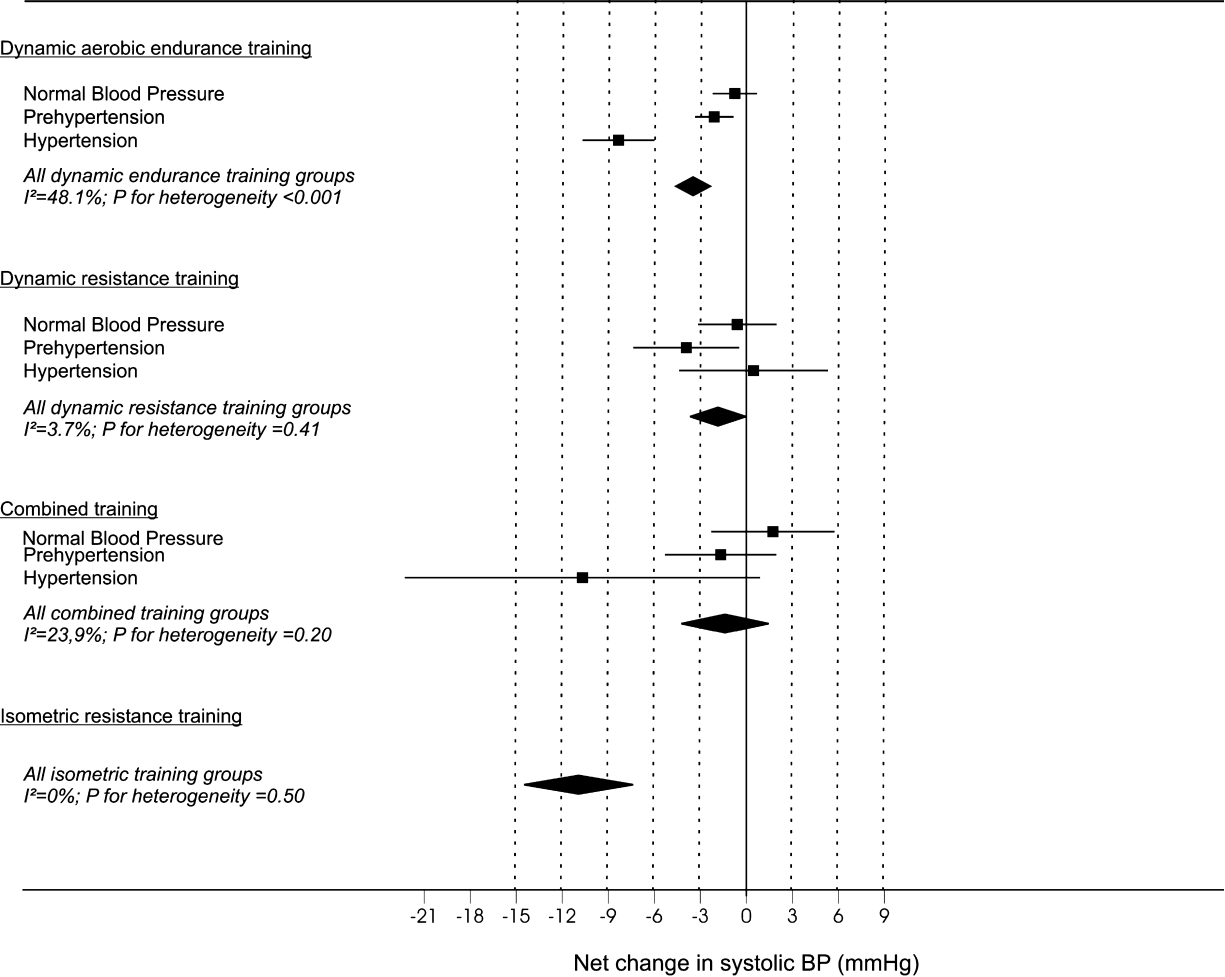
Net changes in systolic blood pressure (BP) after different exercise modalities using random‐effects analyses. Data are reported as net mean changes, adjusted for control data (95% confidence limits).

**Figure 3.**
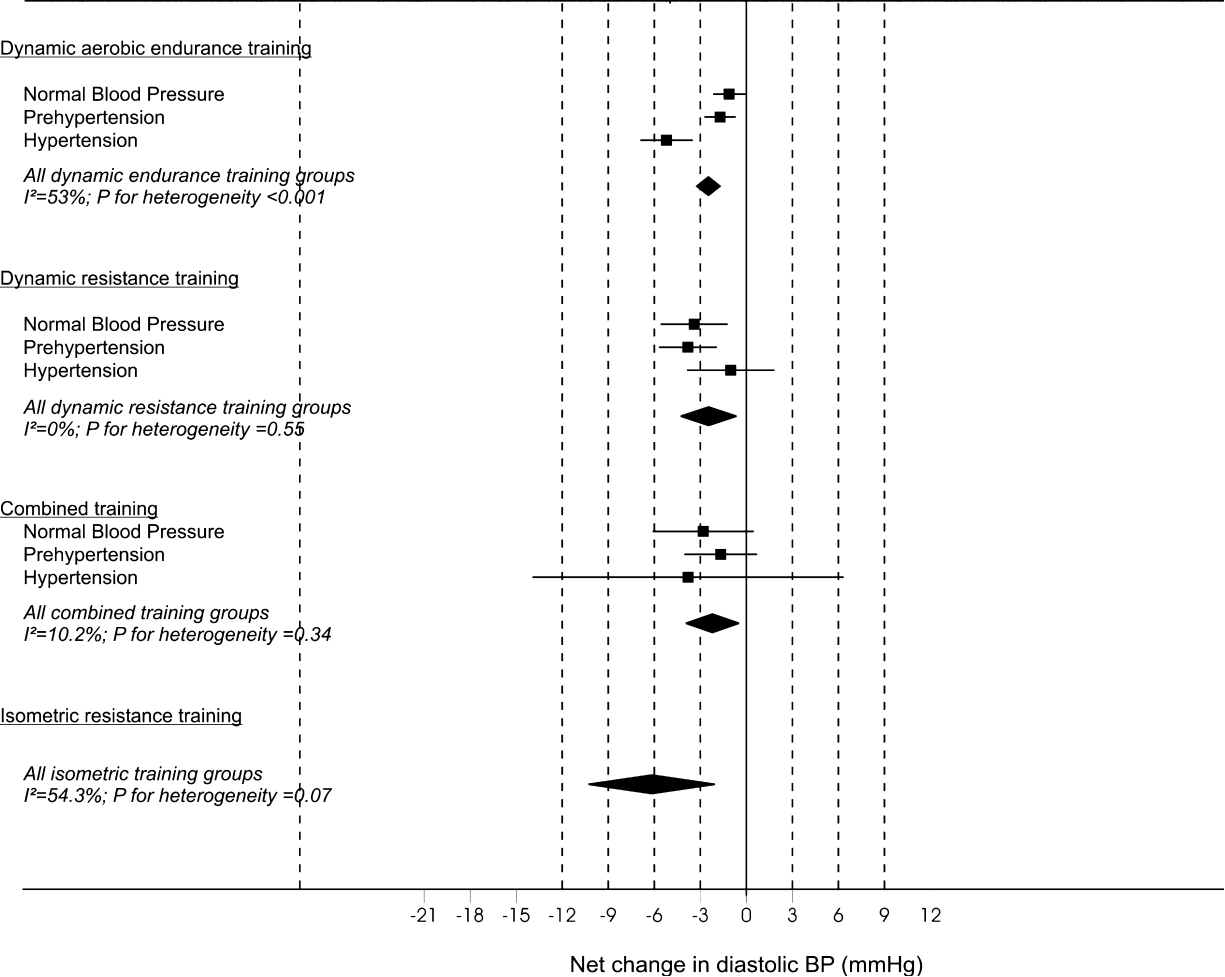
Net changes in diastolic blood pressure (BP) after different exercise modalities using random effects analyses. Data are reported as net mean changes, adjusted for control data (95% confidence limits).

Figures 2 and 3 show the overall results for SBP and DBP. Statistically significant reductions were found for SBP after endurance training (−3.5 mm Hg [−4.6 to −2.3], *P*<0.0001), dynamic resistance training (−1.8 mm Hg [−3.7 to −0.011], *P*=0.049), and isometric resistance training (−10.9 mm Hg [−14.5 to −7.4], *P*<0.0001) but not after combined training (−1.4 mm Hg [−4.2 to +1.5], *P*=0.34). DDBP was significantly reduced after endurance training (−2.5 mm Hg [−3.2 to −1.7], *P*<0.0001), dynamic resistance training (−3.2 mm Hg [−4.5 to −2.0], *P*<0.0001), isometric resistance training (−6.2 mm Hg [−10.3 to −2.0], *P*=0.003), and combined training (−2.2 mm Hg [−3.9 to −0.48], *P*=0.012). Overall, there were no significant differences between the effects of endurance training, dynamic resistance training, and combined training on SBP and DBP (*P*>0.05 for all). Similar, the 10 trials that included both an endurance training and a dynamic resistance training arm showed no significant differences between exercise modalities for SBP (*P*=0.76) and DBP (*P*=0.94) effects. By contrast, reductions in SBP and DBP were larger after isometric resistance training compared with endurance training, dynamic resistance training, or combined training, although they were significant only for SBP (*P*<0.001 for all).

Cumulative meta‐analyses showed that results have remained significant for the effect of endurance training on SBP and DBP since 1985 and 1990, respectively. For dynamic resistance training, cumulative meta‐analysis showed that results remained significant since 2007 for SBP and since 1997 for DBP. Finally, with regard to isometric resistance training, the results have remained highly significant since the first publication in 1992 for both SBP and DBP.

The effect of endurance training on SBP (*P*<0.0001) and DBP (*P*<0.0001) was greatest in 26 study groups with hypertensive participants (−8.3 [−10.7 to −6.0]/−5.2 [−6.9 to −3.4] mm Hg) compared with groups with participants with prehypertension (−4.3 [−7.7 to −0.90]/−1.7 [−2.7 to −0.68] mm Hg) or normal BP (−0.75 [−2.2 to +0.69]/−1.1 [−2.2 to −0.068] mm Hg). The effect of dynamic resistance training on SBP and DBP tended to be greater in prehypertensive individuals although not significant (*P*>0.10).

### Subgroup Analyses

Results of subgroup analyses for endurance training are summarized in Table 1. Subgroup analyses of endurance training suggested that male participants achieved greater than twice the reduction in SBP (*P*<0.01) and DBP (*P*=0.011) as female participants. Program duration of <24 weeks appears to lower SBP (*P*<0.0001) and DBP (*P*<0.01) to a greater extent than programs of >24 weeks’ duration. Lower training intensity is associated with the smallest effect size on SBP (*P*=0.032) and DBP (*P*=0.030). Less than 210 minutes of weekly endurance training showed significantly larger SBP (*P*<0.05) and borderline but not significant DBP (*P*=0.198) reductions. Individual exercise session durations of 30 to 45 minutes showed larger reductions in SBP and DBP, although they were statistically significant only for DBP (*P*<0.001). The following subanalyses suggested no difference between subgroups: age >50 versus <50 years and weekly exercise training frequency. Furthermore, we observed a tendency for larger reductions in SBP (β1=0.49, *P*=0.08) and DBP (β1=0.45; *P*=0.06) with greater reductions in weight after dynamic endurance training.

**Table 1.** Subgroup Analyses for the Effect of Dynamic Endurance Training on Resting BP Using a Random‐Effects Model

	Systolic BP		Diastolic BP	
	N	Effect Size (95% CL)	N	Effect Size (95% CL)
Sex				
Male	28	−4.7 (−2.2 to −1.6)	28	−2.8 (−1.3 to −4.5)
Female	38	−0.87 (−2.2 to +0.46)	35	−0.56 (−1.39 to +0.27)
Age, y				
<50	54	−2.8 (−3.9 to −1.8)	50	−3.0 (−4.3 to −1.7)
≥50	50	−3.9 (−5.9 to −2.0)	52	−1.8 (−2.7 to −1.0)
Hypertensive status				
Normal BP	29	−0.75 (−2.2 to +0.69)	29	−1.1 (−2.2 to −0.068)
Prehypertension	50	−2.1 (−3.3 to −0.83)	47	−1.7 (−2.7 to −0.68)
Hypertension	26	−8.3 (−10.7 to −6.0)	26	−5.2 (−6.9 to −3.4)
Duration of the intervention, wk				
<12	19	−6.4 (−9.9 to −2.9)	17	−4.0 (−6.4 to −1.6)
12 to 24	51	−4.1 (−5.2 to −3.0)	50	−3.0 (−4.0 to −1.9)
>24	35	−0.77 (−1.9 to +0.40)	35	−1.7 (−2.2 to −0.17)
Exercise frequency, times weekly				
<3	11	−3.9 (−7.4 to −0.39)	11	−2.8 (−4.8 to −0.75)
3 or 4	63	−3.5 (−5.0 to −2.0)	63	−2.4 (−3.6 to −1.3)
>4	31	−3.2 (−4.9 to −1.5)	28	−2.4 (−3.4 to −1.4)
Exercise intensity^5^				
Low	7	+0.073 (−2.8 to +2.9)	7	+0.32 (−1.9 to +2.5)
Moderate	32	−4.8 (−7.5 to −2.2)	31	−2.3 (−3.3 to −1.3)
High	57	−3.6 (−4.7 to −2.5)	55	−3.1 (−4.3 to −1.9)
Session duration, min/session				
<30	9	−0.43 (−3.4 to +2.5)	9	+0.62 (−1.0 to +2.3)
30 to 45	55	−3.8 (−4.9 to −2.6)	53	−3.3 (−4.4 to −2.2)
>45	38	−2.8 (−5.0 to −0.62)	38	−1.9 (−3.1 to −0.70)
Weekly exercise time, min/wk				
<150	43	−3.6 (−4.9 to −2.2)	43	−2.7 (−4.0 to −1.4)
150 to 210	46	−3.9 (−5.8 to −2.0)	44	−2.7 (−3.8 to −1.6)
>210	13	+0.2 (−2.3 to +2.8)	13	−0.92 (−2.6 to +0.79)

BP indicates blood pressure; CL, confidence limit; N, number of study groups.

Subgroup analyses for DRT are given in Table 2. SBP and DBP reductions after dynamic resistance training were not significantly different with regard to sex, age category, duration of the exercise program, or training intensity.

**Table 2.** Subgroup Analyses for the Effect of Dynamic Resistance Training on Resting BP Using a Random‐Effects Model

	Systolic BP		Diastolic BP	
	N	Effect Size (95% CL)	N	Effect Size (95% CL)
Sex				
Male	8	−3.9 (−6.9 to −0.91)	8	−0.80 (−3.8 to +2.2)
Female	9	−3.1 (−5.7 to −0.58)	9	−2.6 (−7.0 to +1.76)
Age, y				
<50	13	−0.99 (−3.3 to +1.4)	13	−3.1 (−5.2 to −1.1)
≥50	16	−3.1 (−6.1 to +0.12)	16	−3.4 (−5.3 to −1.6)
Hypertensive status				
Normal BP	12	−0.59 (−3.1 to +2.0)	12	−3.4 (−5.6 to −1.2)
Prehypertension	13	−4.3 (−7.7 to −0.90)	13	−3.8 (−5.7 to −1.9)
Hypertension	4	+0.47 (−4.4 to +5.3)	4	−1.0 (−3.9 to +1.9)
Duration of the intervention, wk				
<12	5	−1.6 (−7.2 to +3.9)	5	−2.3 (−5.8 to +1.2)
12 to 24	18	−2.0 (−4.7 to +0.62)	18	−3.4 (−4.9 to −1.8)
>24	6	−2.6 (−6.9 to +1.8)	6	−3.6 (−6.6 to −0.59)
Exercise intensity^5^				
Low	2	−5.8 (−14.8 to +3.1)	2	−4.7 (−10.4 to +1.0)
Moderate	5	−3.2 (−8.6 to +2.3)	5	−4.5 (−9.5 to +0.46)
High	20	−2.0 (−4.4 to +0.22)	20	−3.0 (−4.6 to −1.5)

BP indicates blood pressure; CL, confidence limit.

### Publication Bias and Sensitivity Analyses

The funnel plots of the primary analyses are shown in Figures S1 through S8. Funnel plots including Egger regression tests (*P*>0.10 for all) for the different analyses did not suggest publication bias, nor did Duval and Tweedie's trim and fill computation change the results.

## Discussion

Our meta‐analysis of published randomized controlled parallel‐design studies of exercise training in subclinical populations is the largest such analysis of exercise training on BP to date, containing data on >5000 participants. Our results demonstrate that endurance training, dynamic resistance training, combined training, and isometric resistance training significantly reduce DBP and all except combined training reduce SBP. Furthermore, this meta‐analysis demonstrates the largest effect sizes are observed after isometric handgrip or leg exercise, but there is a current paucity of published studies that examine this type of intervention. No significant differences in effect size were observed between endurance training and dynamic resistance training, although our analyses suggest that in those with HTN, endurance training might be superior to dynamic resistance training or combined training. Finally, larger BP reductions after endurance training were observed from shorter exercise program durations at moderate to high intensity and <210 minutes of weekly exercise. Collectively, these findings have implications for exercise training prescription and delivery for BP management.

Dynamic endurance training, dynamic resistance training, and combined training were each associated with decreases in SBP and DBP, and magnitudes of these reductions were similar across these 3 exercise modalities. After dynamic endurance training, we found BP decreases were most pronounced in male participants and hypertensive participants, but significant reductions were also observed in participants with normal BP and prehypertension. However, after dynamic resistance training, reductions in SBP and DBP were largest in the study groups of prehypertensive participants. Moreover, the effects of endurance training, dynamic resistance training, and combined training on SBP and DBP in the individual with normal BP or prehypertension were similar, underlining the value of dynamic resistance training as an adjunct therapy for the prevention of high BP in these preclinical populations. Our results suggest endurance training might be superior to dynamic resistance training for hypertensive individuals, although it should be noted only 4 of 29 dynamic resistance study groups involved hypertensive patients. Therefore, until clearer evidence emerges, it may be prudent to prescribe endurance training rather than dynamic resistance training for the hypertensive individual if lower BP is desired.

Our findings further demonstrate that isometric handgrip training and isometric leg training result in larger reductions in SBP and a trend toward lower DBP compared with the 3 other exercise modalities, but the paucity of studies to date limits the strength of this conclusion. As stated earlier, there is no between‐trial heterogeneity among the 5 isometric training groups,^8^ and lack of significant publication bias suggests the findings are robust, although generalizability of the results might be premature as data were available from only 4 trials (5 study groups).

Subgroup analyses of endurance training further demonstrate exercise programs of <6 months induced larger BP reductions compared with programs of longer duration; this concurs with previous meta‐analyses and might be explained by unsupervised exercise sessions, a characteristic of the longer program durations and associated with reduced adherence. Indeed, subgroup analyses of dynamic resistance training showed no difference between programs of shorter and longer durations, perhaps because most trials provided supervised sessions and previous work shows facility‐based exercise programs yield the highest adherence rates.^19^

BP reductions after low‐intensity endurance training (<40% heart rate reserve or <55% heart rate maximum) were smaller compared with moderate‐ or high‐intensity training, and no significant differences in BP responses were observed between low‐, moderate‐, and high‐intensity dynamic resistance training. This might be explained by the fact that most dynamic resistance training study groups exercise at higher intensity (>69% 1‐repetition maximum), with only 7 dynamic resistance groups performing at low intensity. Training frequency and exercise session duration did not significantly affect the BP response to endurance training, but >210 minutes of weekly exercise produced the smallest reductions in BP, which appears counterintuitive as one would presume exercise training‐induced BP reductions follow a dose–response relationship. One possible explanation might be that programs with a total of >210 minutes per week are performed at lower intensity. Given that multivariable analyses were not possible we took into consideration that the overall recommendation is that higher volume (product of intensity, frequency, and duration) is associated with larger health benefits. Finally, after endurance training, we observed a tendency for larger changes in BP associated with larger reductions in weight. Although this relation did not reach statistical significance, the observed trend does not exclude a causal role in the BP response to training because of the many differences between study groups and the multiple potential mechanisms involved in the BP response.

### Limitations

A number of potential limitations of the current meta‐analysis have to be considered. First, there are limitations inherent to the primary literature. (1) Participants are aware of their allocation to a control or intervention group in exercise studies. (2) Several important scientific criteria have not always been observed, such as regular follow‐up of the control subjects, assessment of compliance to the training program, attention to changes in other lifestyle factors, and lack of blinded or automated measurements. The small number of studies that conducted an intent‐to‐treat analysis makes it impossible to quantify the impact of study withdrawals. With regard to the latter, it is recommended that future studies report both per‐protocol and intent‐to‐treat analyses so that one can determine both the efficacy and effectiveness of the different interventions on BP. (3) To examine dose–response effects, additional information on energy expenditure or a detailed description of the duration of each exercise at a given intensity should be provided in future studies.

Other limitations are associated with the meta‐analytic technique itself as it should be acknowledged that meta‐analysis is no substitute for large well‐designed randomized controlled trials. However, the meta‐analytical technique is probably the best method currently available to systematically review previous work.^20^ Advantages are the greater precision of the estimates and the enhanced statistical power. Potential disadvantages included the heterogeneity of studies and potential publication bias. Nevertheless, despite strict selection criteria, studies may differ in several respects, but this potential problem is addressed by applying a random‐effects models and by exploring the heterogeneity and inconsistency of studies. Furthermore, analyses of asymmetry of the funnel plot by Begg and Egger test did not suggest any publication bias. A final potential limitation is the large number of statistical tests that were conducted in this meta‐analysis. As a result, some of the significant findings could have been merely chance. However, as suggested by others,^21–22^ adjustments for multiple tests were not made because of the problems associated with such. Furthermore, given that all of our conclusions are based on *P* values <0.01, we hypothesize that risk for type I error is low. Nevertheless, findings based on meta‐analyses always need to be confirmed with large, well‐designed randomized controlled trials.
